# Cone-Beam Computed Tomography Assessment of Prevalence of Procedural Errors in Maxillary Posterior Teeth

**DOI:** 10.1155/2023/4439890

**Published:** 2023-12-12

**Authors:** Ahmad Nouroloyouni, Yousef Nazi, Hesam Mikaieli Xiavi, Sara Noorolouny, Maryam Kuzekanani, Gianluca Plotino, James Laurence Walsh, Behzad Sheikhfaal, Rashin Alyali, Elham Tavakkol

**Affiliations:** ^1^Department of Endodontics, School of Dentistry, Ardabil University of Medical Science, Ardabil, Iran; ^2^Department of Radiology, Ardabil University of Medical Sciences, Ardabil, Iran; ^3^Department of Pediatric Dentistry, School of Dentistry, Ardabil University of Medical Science, Ardabil, Iran; ^4^Endodontology Research Center, Department Of Endodontics, School of Dentistry, Kerman University of Medical Sciences, Kerman, Iran; ^5^Private Practice, Grande Plotino & Torsello Studio di Odontoiatria, 00187 Rome, Italy; ^6^UQ Oral Health Centre, School of Dentistry, The University of Queensland, Brisbane, Australia; ^7^Department of Endodontics, School of Dentistry, Tabriz University of Medical Science, Tabriz, Iran; ^8^Department of Radiology, Guilan University of Medical Sciences, Rasht, Iran; ^9^Department of Radiological Sciences, UCLA, Los Angeles, CA, USA

## Abstract

A range of procedural errors can occur when performing endodontic treatment on posterior teeth. These errors may decrease the success rate in endodontic practice. This study assessed the prevalence of procedural errors and the quality of endodontic treatments in maxillary molars and premolars using cone-beam computed tomography (CBCT). CBCT scans from two private radiology centers were assessed retrospectively to ensure the same calculated sample size of 327 teeth for each of the four maxillary posterior tooth types (a total of 1,308 endodontically treated teeth). Image sets were evaluated for procedural errors categorized as follows: obturation length (overfilling or underfilling by >2 mm short of the root apex), missed canals, perforations, strip perforations (with extrusion of material into the furcation area), separated instruments in the root canal space, and root fracture. Data were analyzed with SPSS version 20 (SPSS Inc., Chicago, IL, USA), and frequency data was assessed using the Monte Carlo test at the 0.05 level of significance. The procedural errors most commonly reported in the present study were from most frequent to least frequent: underfilled canals (50.0%), missed canals (27.5%), overfilled canals (12.5%), apical perforations (5.0%), separated instruments (3.1%), and root fractures (1.9%). No strip perforations (with extrusion of material into the furcation area) were seen in the study (0%). Underfilled and missed root canals were the most frequent procedural errors identified in the present study. These findings underline the importance of more consideration of critical working length management during all stages of root canal treatment, greater awareness of root canal anatomy, and the use of imaging and diagnostic devices that enhance the ability to identify and treat root canals both safely and effectively.

## 1. Introduction

The success of root canal treatment (RCT) can be compromised by a range of procedural errors, leading to persistent periapical disease [1, 2]. Intraoperative procedural errors need to be prevented and recognized by clinicians when they occur in order to improve the prognosis of RCT [3].

The quality of RCT and incidence of procedural errors can vary between general dentists and endodontists and also among different regions, as documented in several studies [4–8]. Procedural errors can include missed canals (detecting an unfilled root canal by changing the radiographic angle), over- or underfilled canals (overfilling beyond the tooth apex, underfilling by >2 mm short of the root apex), and root perforations (communication between the root canal system and periodontium), as well as instrument separation (presence of a broken piece of instrument in the root canal space or in the periapical area) [9]. Previous studies have reported that the most frequent procedural errors were ledges [10], underfilled canals [6, 11], and inadequate radiopacity of the root filling [12].

These procedural errors have been clearly demonstrated to negatively impact treatment success. Studies show that underfilling the root canal reduces success rates to just 68%, while overfilling decreases success to 76% [13–17]. Likewise, cases involving instrument separation during treatment have 14% lower success rates compared to those without this error [13, 17]. The evidence overwhelmingly indicates that certain mistakes like inadequate filling and separated instruments substantially lower the chances of a favorable outcome. Preventing such errors must be a top priority when doing root canal therapy in order to maximize the likelihood of success.

Past works on procedural errors have typically used conventional two-dimensional radiographs [4–8, 11, 12]. To overcome the limitations of this approach, in this study, a trained and calibrated senior dental student evaluated the endodontically treated maxillary molars and premolars on CBCT scans under the supervision of an oral and maxillofacial radiologist and an endodontist. The teeth were evaluated using cone-beam computed tomography (CBCT) images in the sagittal, coronal, and axial planes to provide a three-dimensional evaluation of the treated teeth without distortion, overlapping, or superimposition of anatomical structures [18].

CBCT has been used for evaluating the morphology of teeth with complex root canal anatomy [19, 20], but studies to assess the presence of procedural errors in root canal-treated maxillary posterior teeth are very limited [21].

Special care should be taken when working on maxillary posterior teeth, which had a significantly higher error rate when compared to anterior teeth [1]. Some studies have examined the prevalence of iatrogenic errors involving mandibular premolars and molars in our studied population [6, 22]. However, there is also a high frequency of errors in maxillary first molars and other posterior teeth, yet information about them is very limited and has mostly involved root canals performed by dental students and two-dimensional radiographs [5, 7, 8, 11]. By adding this information to the literature, we can identify the most frequent errors and use this knowledge to achieve target-based continuing education in order to address the most critical gaps [6]. This paper examined root canal treatments across an entire population, reporting problems encountered throughout the dentistry community. As such, it provides valuable information that could be used to guide targeted continuing education and improve dentists' work after graduation.

As a consequence, the aim of the present study was to evaluate CBCT scans to report on the presence of different procedural errors that occurred during RCT in maxillary posterior teeth.

## 2. Materials and Methods

The present study was approved by the ethics committee of the Ardabil University of Medical Sciences (IR.ARUMS.REC.1398.397). CBCT scans from the two private radiology centers in Ardabil were assessed retrospectively. Those two private radiology centers were the only centers with CBCT imaging in the Ardabil state (studied population). The patients consented to the use of their CBCT scans for research purposes at the time of radiography. The CBCT scans had been taken for diagnostic and treatment planning purposes not related to this study.

The sample size was 327 for each of the four maxillary tooth types (first and second premolars and molars), as calculated using the Krejcie and Morgan sample size equation as there were 2180 CBCTs available. On the other hand, there was no similar study using CBCT for evaluating iatrogenic errors when conducting the study, giving a total of 1,308 teeth. The scans from 780 patients were analyzed to meet the sample size requirements for endodontically treated teeth. Any teeth that had metal posts were excluded due to severe artifacts which could cause inaccurate interpretation of the radiographs.

Both radiology centers used the same CBCT system (ProMax® 3D Mid, Planmeca, Helsinki, Finland) with a voxel size of 75 *μ*m. Romexis® Viewer version 6.0 software was used to evaluate the images. A trained and calibrated senior dental student evaluated the endodontically treated maxillary molars and premolars on CBCT scans under the supervision of an oral and maxillofacial radiologist and an endodontist. The teeth on CBCT images were evaluated in the sagittal, coronal, and axial planes in terms of obturation length (overfilling beyond the tooth apex, underfilling by >2 mm short of the root apex), perforation (communication between the root canal system and periodontium), number of missed root canals (detecting an unfilled root canal by changing the radiographic angle), strip perforation (noticing the extrusion of sealer or gutta-percha from the root canal wall into the furcation area in multirooted teeth), broken instruments (presence of a broken piece of instrument in the root canal space or in the periapical area), and root fracture. To ensure the validity of the assessments, 25% of the CBCT scans were randomly selected and reevaluated by the oral and maxillofacial radiologist and endodontist. The interexaminer agreement between the findings was assessed using Cohen's kappa, which resulted in a fully reliable agreement (kappa = 0.9). Also, to assess intraexaminer reliability, all CBCT scans were revaluated by the senior dental student 10 days after their primary assessment, and the agreement between the findings in the first and second observations was calculated, yielding 100% agreement (kappa = 1).

## 3. Statistical Analysis

Data were analyzed with SPSS version 20 (SPSS Inc., Chicago, IL, USA), and given the objectives of the study and the nature of the variables under investigation (which are nominal) and also the fact that more than 47% of the cells in the contingency table had frequencies less than 5, the Monte Carlo test was used to examine the association between the nominal variables under study at the 0.05 level of significance.

## 4. Results

The distribution of procedural errors is summarized in Tables 1–3.

Underfilling was most common in the mesiobuccal root of maxillary first molars and was more common in molars than premolars (*P* < 0.05).

The most frequently missed root canal was the second mesiobuccal root canal (MB2) of the maxillary first molar. Overall, missed root canals were more common in molars than premolars (*P* < 0.05).

The most common site for finding separated instruments was the first mesiobuccal root canal in maxillary first molars, while the most common sites for apical perforation were the buccal root canal of second premolars and palatal and the distobuccal root canals of maxillary first molars. Overall, separated instruments were more common in premolars than molars (*P* < 0.05), while apical perforations were more common in molars (*P* < 0.05).

Root fractures were seen only in the palatal root of maxillary first premolar teeth, and no cases of strip perforations were detected.

## 5. Discussion

The present study evaluated procedural errors in the maxillary premolar and molar teeth using CBCT in a selected Middle Eastern population. Ethnicity and geographic region can influence root canal anatomy and impact endodontic procedural errors in several ways. Tooth morphology can vary between different ethnic populations [23]. For example, studies have shown that Asian populations have a higher prevalence of single-rooted maxillary first premolars compared to other ethnicities [24]. Maxillary first and second molars with a second mesiobuccal canal were more commonly found in whites compared to Asians (71.3% vs. 58.4% and 58.5% vs. 31.6%, respectively) [24]. These anatomical variations mean clinicians must be prepared for a wider range of possible morphologies in certain regions/ethnic groups. Lack of knowledge of population-specific anatomy raises the risk of procedural errors [25]. For instance, overzealous searching for a second canal in the mandibular first molars of Asian patients can lead to perforation, while a lack of awareness of extra canals could result in missed anatomy.

Overall, ethnicity and geography influence anatomy which must be appreciated to avoid procedural mistakes. Customizing techniques for the population treated and having sound knowledge of regional morphologic variations are key to preventing errors and improving outcomes.

Cone-beam computed tomography (CBCT) enables three-dimensional evaluation of the root canal anatomy and quality of root canal treatments and eliminates the limitations of two-dimensional images. CBCT enables undistorted three-dimensional visualization of the dentition without distortion, overlapping, and superimposition of anatomical structures [18]. CBCT is more valuable for the evaluation of the morphology of teeth with complex root canal structures and the quality of their RCT [19, 20].

## 6. Underfilling

The present study showed that a range of procedural errors occurred that could readily be detected using CBCT imaging. The overall patterns reported through CBCT evaluation in the present study reinforce the findings of previous studies based on periapical radiographs, with underfilling being the most common procedural error, especially in the MB1 canal of maxillary first molars [10, 12, 26–33]. Underfilling of root canals in maxillary molars can be explained by their more complex anatomy with narrow and curved root canals and an increased probability of ledge formation compared to maxillary premolars [29]. Discrepancies between the present results and some previous studies [34, 35] might reflect differences in the training and expertise of clinicians, as well as different methodologies of evaluation. Alrahabi [34] evaluated the root canal treatments done by undergraduate students using periapical images, and Madfa et al. [35] evaluated the root canal treatments done by general dentists using panoramic images. In the study of Alrahabi, the prevalence of underfilling was less than that of overfilling in maxillary molars. The higher rate of underfilling observed in the present study compared to the study of Alrahabi may be attributed to methodological differences. This study examined root canal treatments performed in a general population, including experienced dentists and endodontists, whereas Alrahabi focused on undergraduate dental students. Practicing clinicians would be expected to have superior skills in maintaining working length and apical stops compared to students. Additionally, the 3D nature of CBCT allowed for enhanced detection of underfilling due to better visualization of buccal and palatal curvature in the apical third of canals, which can be challenging to appreciate on 2D radiographs. The advanced imaging and more representative sample likely contributed to the higher underfilling prevalence observed in this study population.

## 7. Overfilling

In the present study, the highest rate of overfilling occurred in the maxillary second premolars. This could reflect the fact that the apical foramen in single-canal maxillary second premolars may be wide, which makes it more difficult to achieve a good seal [36]. On the other hand, the frequency of overfilling was more in maxillary molars in comparison with maxillary premolars. Overall, the present results for overfilling are consistent with a wider range of previously published studies [10, 12, 27–32, 34, 35]. In addition to the posterior location of maxillary molars, which makes it more difficult to treat, errors in determining the anatomy of maxillary posterior teeth can occur in periapical radiographs because of the superimposition of the floor of the maxillary sinus and the maxillary process of the zygomatic bone [37].

Khabbaz et al. [33] reported less frequency of overfilling in maxillary molars than in premolars. This difference might be due to either superimposition of the maxillary process of zygomatic bone in periapical radiographs used to evaluate the data, and this might lead to misinterpretation of data or a difference in the skills of expertise of clinicians since in the mentioned study undergraduate students performed the treatments, in which usually premolars were treated by juniors and molars by senior students.

### 7.1. Missed Canal

In the present study, the most frequently missed root canal was the MB2 canal of maxillary first molars. Missed root canals were more frequent in the maxillary molars than premolars, consistent with the results of a study by Moradi et al. [29] Missed anatomy is one of the main causes of endodontic failure. An untreated canal space represents a reservoir for microorganisms and may cause endodontic treatment failures [38]. Understanding molar root canal anatomy and using appropriate magnification are essential for locating and instrumenting MB2 canals [39]. The orifice of the MB2 canal is located in a mesiopalatal position with respect to that of the MB1 canal. As a consequence, it is more challenging to detect and negotiate and hence more likely to be missed [36, 40]. In addition, several variations in the root canal system of maxillary molars have also been reported [41–43], and the prevalence of MB2 canals differs based on patient ethnicity [44], with a global average of 74% [45]. The known prevalence for the MB2 canal in an Iranian population was 86.6% and 67.5% in the first and second maxillary molars, respectively [46, 47]. In maxillary premolars, the highest frequency of missed canals occurred in the palatal canal of the second premolars. The prevalence of missed roots in these teeth may be attributable to an insufficient understanding of the potential for two canals being present, leading clinicians to assume they are single-rooted [48]. Further education on techniques for finding MB2 in maxillary molars and the variable anatomy of maxillary second premolars appears warranted to ensure clinicians are aware these teeth can frequently contain an additional canal that must be located and treated.

### 7.2. Separated Instrument

The results of the present study indicated that separated instruments were found mostly in the MB1 canal of maxillary first molars. This finding is consistent with previous investigations [27–32]. A higher rate of separated instruments in first molars than in premolars can be explained by their more difficult access as well as the presence of greater anatomical complexities [29]. Other contributing factors include the instrument type and design used, as well as operator experience and decision-making around how many times to reuse the same instrument [49–51].

In contrast to our findings, AlRahab [34] reported a higher rate of broken instruments in maxillary premolars compared to molars. Multiple factors can contribute to instrument fractures including clinician experience, instrument type, and design [36, 38].

According to Parashos et al. [50], the operator and their clinical skills are the most important determinants of instrument fracture, beyond conscious decisions about reuse. Furthermore, Yared et al. [51] found more experienced clinicians had lower fracture rates. In AlRahabi's study, undergraduate students performed the treatments, with premolars done by juniors and molars by senior students. This highlights the critical role of expertise in minimizing file fractures, beyond the influence of tooth anatomy.

## 8. Perforation

Apical perforations were detected in the buccal root canals of maxillary second premolars and the palatal and distobuccal root canals of maxillary first molar teeth. This finding is in agreement with previously published studies [27–29, 32, 34, 52]. However, some studies, including those by AlRahabi [34] and Akbar [32], reported no significant difference in the number of apical perforation errors between maxillary molars and premolars. Haji-Hassani et al. [28] reported a higher frequency of apical perforation in maxillary premolars than in molars. The difference between Haji-Hassani et al. [28] and the present study is due to different methodologies, since in Haji-Hasani's study, overfilled teeth were also considered apical perforation.

Strip perforations were not detected in the present study. Consistent with these findings, Akbar [32] reported no strip perforation in maxillary molar and premolar teeth. However, studies by Dadresanfar et al. [10], Haji-Hassani et al. [28], and Moradi et al. [29] showed that strip perforation was more frequent in maxillary molars than premolars. Moradi et al. [29] also mention that the low frequency of this perforation might be due to the fact that they are evaluating root canal treatments done by undergraduate students, and strip perforations are mostly referred to postgraduation students after the occurrence. In the present study, no strip perforation was detected, which might be attributed to the poor prognosis of such teeth, leading to their extraction.

## 9. Root Fracture

Root fractures were found at a low prevalence in the palatal root of the first premolars. This is consistent with previous work [53–55] and may reflect a greater susceptibility to fracture for this root due to intrinsic points of weakness and high occlusal loading [56]. Root fractures are challenging to detect on conventional radiographs [53] but can readily be seen by using CBCT imaging [57–61]. The exclusion of teeth with metallic posts can also be a reason for the low prevalence of root fractures reported in this study. Biomechanical experiments revealed that high tensile stresses and regions of stress concentrations in the remaining dentin structure result in increased predilection of VRFs in teeth with the post [62].

## 10. Limitations

The limitations of the present study include the details of the types of clinicians who undertook RCT (including their training, level of experience, and techniques used), which could not be determined, and the interval between RCT and CBCT examination, which was also unknown. For these reasons, contributing factors to the iatrogenic errors reported in the present study were not able to be explored.

Another major limitation of the current study is that, due to the lower prognosis of teeth with iatrogenic errors especially teeth with vertical root fractures and perforations, it appears that the actual prevalence should be higher than what was reported in the present study. According to Almasri [63], the most frequently extracted teeth were molars (55.6%), followed by premolars (30.3%) in their study, and 25.3% of extractions were due to vertical root fracture. Ng et al. [64] reported that perforations have significant adverse effects on the survival of endodontically treated teeth. As a result, the reported rate of iatrogenic errors in the present study is probably an underestimation of the actual rate.

Finally, the exclusion of teeth with metallic posts due to artifacts can cause fewer reports of some errors like root fractures and perforations.

## 11. Conclusion

Underfilled and missed root canals were the most frequent procedural errors identified in the present study in maxillary molars and premolars using CBCT as an evaluation system. These findings underline the importance of working length management during all stages of root canal treatment and also greater awareness of the root canal anatomy, along with the routine use of magnification and diagnostic systems that enhance the ability of the operator to identify and treat root canals both safely and effectively.

## 12. Recommendation

Considering the results of the current study, performing targeted continuing education courses about the following topics has the highest priority:

1. Working length determination and maintenance, especially focusing on new technologies like electric apex locators which will reduce working length-related errors
2. Workshops on dental operating microscope which has significant help in finding extra canals
3. Workshops about CBCT images which act as a map in complex cases to reduce missed and perforated canals in addition to reducing unexpected curvatures and file separations
4. Comprehensive anatomical courses based on related geographical and ethnic papers

## 13. Recommendation for Future Researches

Further, large-scale multicenter studies are recommended to compare procedural error frequencies on a broader level, including between general dentists and endodontists, different genders, and age groups.

Long-term investigations examining the impact of educational interventions and equipment advancements on error rates are warranted.

Studies on techniques and equipment to reduce metallic postinduced artifacts in CBCT images are also recommended.

## Figures and Tables

**Table 1:** Procedural errors in maxillary premolar teeth.

Tooth	Maxillary first premolars (*n* = 327)		Maxillary second premolars (*n* = 327)		
Root canal	P	B	P	B	Single canal
Root fracture	2 (6.9%)	1 (3.4%)	0	0	0
Apical perforation	1 (3.4%)	0	0	2 (9.5%)	0
Strip perforations (with extrusion of material into the furcation area)	0	0	0	0	0
Broken instrument	1 (3.4%)	1 (3.4%)	0	1 (4.8%)	0
Missed canal	2 (6.9%)	0	7 (33.3%)	0	0
Underfilled	14 (48.3%)	6 (20.7%)	0	4 (1.0%)	0
Overfilled	0	1 (3.4%)	0	0	7 (33.3%)

P = palatal; B = buccal.

**Table 2:** Procedural errors in maxillary molar teeth.

Tooth	First molars (*n* = 327)				Second molars (*n* = 327)			
Root canal	MB1	MB2	P	DB	MB1	MB2	P	DB
Apical perforation	0	0	2 (2.1%)	2 (2.1%)	0	0	0	1 (5.9%)
Strip perforations (with extrusion of material into the furcation area)	0	0	0	0	0	0	0	0
Broken instrument within the root canal	3 (3.2%)	0	0	1 (1.1%)	0	0	0	0
Missed root canal	0	31 (32.6%)	0	0	0	0	0	4 (23.5%)
Underfilling	40 (42.1%)	4 (4.2%)	2 (2.1%)	2 (2.1%)	6 (35.3%)	0	2 (11.8%)	0
Overfilling	0	0	2 (2.1%)	6 (6.3%)	0	0	2 (11.8%)	2 (11.8%)

MB1: mesiobuccal 1; MB2: mesiobuccal 2; DB: distobuccal; P: palatal.

**Table 3:** Comparison of procedural errors between maxillary premolar and molar teeth.

Procedural errors	Total in		Total	Monte Carlo		
	Premolars	Molars		*P* value	95% confidence interval	
					Lower bound	Upper bound
Apical perforation	3 (1.9%)	5 (3.1%)	8 (5.0%)	0.031	0.028	0.034
Broken instrument	3 (1.9%)	2 (1.2%)	5 (3.1%)			
Missed canal	9 (5.6%)	35 (21.9%)	44 (27.5%)			
Underfilled	24 (15.0%)	56 (35.0%)	80 (50.0%)			
Overfilled	8 (5.0%)	12 (7.5%)	20 (12.5%)			
Root fracture	3 (1.9%)	0	3 (1.9%)			
	50 (31.2%)	110 (68.8%)	160 (100.0%)			

Significant differences at *P* < 0.05.

## Data Availability

The data used to support the findings of this study were supplied by the corresponding author under license, and data will be available on request. Requests for access to these data should be made to the corresponding author before 12 months from publication.
